# Easily Applicable Superhydrophobic Composite Coating with Improved Corrosion Resistance and Delayed Icing Properties

**DOI:** 10.3390/polym16192800

**Published:** 2024-10-03

**Authors:** Binbin Zhang, Lixia Zhao, Baorong Hou

**Affiliations:** 1 Key Laboratory of Advanced Marine Materials, Key Laboratory of Marine Environmental Corrosion and Bio-Fouling, Institute of Oceanology, Chinese Academy of Sciences, Qingdao 266071, China; lixia.zhao@qdio.ac.cn (L.Z.); brhou@qdio.ac.cn (B.H.); 2 University of Chinese Academy of Sciences, Beijing 100049, China

**Keywords:** superhydrophobic, aluminum alloy, anti-corrosion, anti-icing, spray-coating

## Abstract

Mitigating the adverse effects of corrosion failure and low-temperature icing on aluminum (Al) alloy materials poses significant research challenges. The facile fabrication of bioinspired superhydrophobic materials offers a promising solution to the issues of corrosion and icing. In this study, we utilized laboratory-collected candle soot (CS), hydrophobic fumed SiO_2_, and epoxy resin (EP) to create a HF-SiO_2_@CS@EP superhydrophobic coating on Al alloy surfaces using a spray-coating technique. Various characterization techniques, including contact angle meter, high-speed camera, FE-SEM, EDS, FTIR, and XPS, were employed to investigate surface wettability, morphologies, and chemical compositions. Moreover, a 3.5 wt.% NaCl solution was used as a corrosive medium to evaluate the corrosion resistance of the uncoated and coated samples. The results show that the capacitive arc radius, charge transfer resistance, and low-frequency modulus of the coated Al alloy significantly increased, while the corrosion potential (*E*_corr_) shifted positively and the corrosion current (*I*_corr_) decreased by two orders of magnitude, indicating improved corrosion resistance. Additionally, an investigation of ice formation on the coated Al alloy at −10 °C revealed that the freezing time was 4.75 times longer and the ice adhesion strength was one-fifth of the uncoated Al alloy substrate, demonstrating superior delayed icing and reduced ice adhesion strength performance.

## 1. Introduction

The 5xxx series aluminum alloys exhibit exceptional welding capabilities, favorable formability, and lightweight and high-strength characteristics, making them widely utilized in various fields such as marine vessels, aerospace, transportation, machinery manufacturing, sports equipment, and architectural structures [1,2]. However, in practical applications, 5xxx series aluminum alloys are prone to corrosion and low-temperature icing, which pose significant risks to their safety and reliability during service. For instance, critical aluminum alloy components in aircrafts are susceptible to localized corrosion and stress corrosion cracking in atmospheric environments, leading to reduced structural integrity, increased maintenance costs, and compromised flight safety. Similarly, aluminum components on ships are easily corroded by chloride ions in marine environments, diminishing navigational safety. In cold climates, aluminum alloy materials used in power transmission lines and towers are prone to low-temperature icing, resulting in power outages or equipment failures, thereby reducing the durability and lifespan of electrical facilities. In the context of wind energy generation, icing on wind turbine blades at low temperatures can form ice layers on the surface, shifting the center of gravity of the blades, affecting power generation efficiency, and potentially causing blade damage [3,4].

The occurrence of corrosion and low-temperature icing ultimately results from interactions at the material–environment interface. Mitigating the corrosion failure and low-temperature icing issues of aluminum alloys has been a primary focus for researchers and industry professionals. Various materials and technologies have been developed for anti-corrosion and anti-icing, including elemental alloying for corrosion protection [5,6], surface oxidation anti-corrosion treatments [7,8], protective anti-corrosion coatings [9,10], thermo-mechanical de-icing [11,12], electrical heating de-icing [13,14], and the addition of de-icing chemical agents [15,16]. However, the design and development of materials that possess both corrosion-resistant and anti-icing properties have always been a great challenge and hold significant practical value.

Wettability is one of the critical properties of solid material surfaces. By modulating surface wettability from hydrophilic to superhydrophobic, it is possible to effectively reduce contact between the environmental medium and the surface, thus providing promising technological solutions for addressing corrosion failure and low-temperature icing problems. Inspired by the unique non-wetting phenomena observed in natural surfaces such as lotus leaves [17], water strider legs [18], and mosquito compound eyes [19], superhydrophobic materials have demonstrated immense application potential across various fields, including anti-corrosion [20], anti-icing [21], anti-bacterial [22], self-cleaning [23], drag reduction [24], oil–water separation [25], energy harvesting [26], droplet manipulation [27], and sensors [28].

Numerous researchers have made significant scientific advancements in the field of superhydrophobic materials for anti-corrosion and anti-icing applications. For example, Zhu et al. [29] prepared a ZIF-7@ZnG@PFDS/EP superhydrophobic coating with a contact angle of 164° and a sliding angle of 3.7° and studied its self-cleaning, anti-icing, and anti-corrosion properties. After 36 days of immersion in a 3.5 wt.% NaCl solution, the impedance modulus of the coating was three orders of magnitude greater than that of the EP coating. The water droplet icing results demonstrate that the coated sample can extend the freezing time of a water droplet from 60 s to 150 s, while the adhesion of ice is 90% lower compared to that of bare glass. Shu et al. [30] manufactured a robust micro-nano-binary structured superhydrophobic coating combining nanosecond laser processing technology and the sol–gel method. The electrochemical test results show that the corrosion current density of the coated substrate decreased by one order of magnitude, with the corrosion inhibition efficiency reaching 90.57%. Moreover, the delayed freezing time of the water column on the coated substrate is 3.33 times longer than that of the normal surface, showing excellent anti-icing performance. Han et al. [31] reported a superhydrophobic TiO_2_/SiO_2_–silane composite film on aluminum alloy through anodization followed by chemical deposition and spin-coating methods. A reduction of two orders of magnitude in corrosion current density and an anti-corrosion efficiency of 99.2% were realized. In addition, the water droplet freezing process of the coated Al alloy sample was remarkably slowed down, indicating good anti-icing performance. However, the current preparation processes for research on superhydrophobic anti-corrosion and anti-icing materials are relatively complicated and mostly focus on evaluating one specific property. Therefore, finding a simple and efficient preparation method that simultaneously evaluates both anti-corrosion and anti-icing performance is a key focus for research and development. Over the past few years, my research team has conducted systematic studies on the design and preparation of bioinspired superhydrophobic materials and their anti-corrosion and anti-icing performance [32,33,34,35,36]. In our research findings, we discovered that spray-coating is a highly convenient and efficient technique for constructing superhydrophobic materials with anti-corrosion and anti-icing properties, offering advantages such as low cost, ease of application, strong substrate compatibility, and suitability for large-scale preparation.

Thus, in this study, we employed a facile spray-coating technology to fabricate a superhydrophobic composite coating on 5083 aluminum alloy substrates. The coating consists of candle soot, hydrophobic fumed SiO_2_, and epoxy resin. Then, we characterized the surface wettability, micro-/nano-binary scale morphologies, and chemical compositions. In addition, the electrochemical behaviors (electrochemical impedance spectroscopy and potentiodynamic polarization curves) and water droplet icing process were investigated to reveal the anti-corrosion and anti-icing properties of the fabricated superhydrophobic composite coating.

## 2. Experimental Section

### 2.1. Materials and Reagents

Hydrophobic fumed SiO_2_ nanoparticles (HF-SiO_2_ NPs, R972, 16 nm, >99.8%) were bought from Degussa AG, Essen, Germany. Ethanol absolute (C_2_H_6_O, >99.7%), sodium chloride (NaCl, >99.5%), and methylene blue trihydrate (C_16_H_18_ClN_3_S·3H_2_O) were bought from Sinopharm Chemical Reagent Co., Ltd. Beijing, China. Candles (main composition: C_25_H_52_) were purchased from Alibaba. Epoxy resin (EP) and its curing agent were supplied by Shenzhen Jitian Chemical Co., Ltd, Shenzhen, China. 5083 aluminum (Al) alloy plates were purchased from Shandong Shengxin Technology Co., Ltd. (Binzhou, China), with a size of 50 × 25 × 1 mm^3^. All reagents in this research study were used as received without further treatment.

### 2.2. Collection of Candle Soot (CS) Particles

A candle was ignited, and after the flame stabilized, a metal plate was fixed above the flame. The height of the metal plate was adjusted to ensure contact with the outer flame, facilitating the efficient collection of candle soot (CS) particles. After a period of burning, a significant amount of black residue covered the metal plate. These deposits were gently scraped off, and the collected CS particles were stored in a desiccator before usage.

### 2.3. Fabrication of Superhydrophobic Coating

In total, 0.1 g of HF-SiO_2_ and 0.2 g of CS particles were added to 20 mL of anhydrous ethanol. The mixture was homogenized and dispersed uniformly using ultrasonic and magnetic stirring, resulting in a stable HF-SiO_2_@CS/ethanol suspension. Subsequently, 0.3 g of epoxy resin and 0.1 g of a curing agent were incorporated into the suspension, which was mixed vigorously for 1 h under strong magnetic stirring to obtain a sprayable HF-SiO_2_@CS@EP/ethanol suspension.

**Figure 1:**
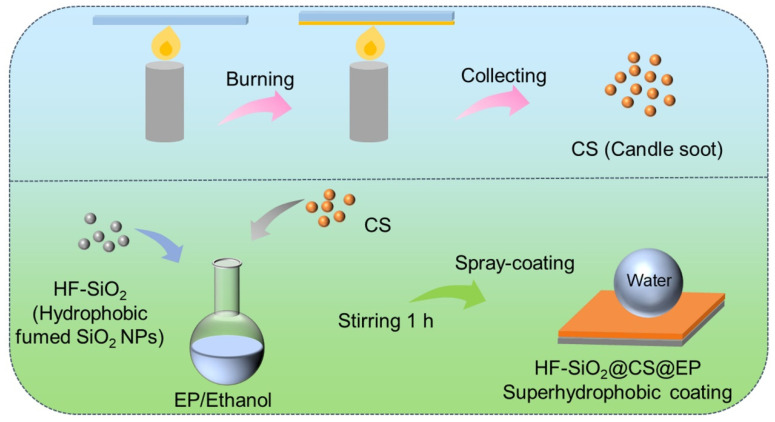
Schematic illustration of the experimental fabrication process for the candle soot particles and superhydrophobic composite coating.

Prior to spraying, the 5083 Al alloy samples were polished using SiC sandpaper of varying grits (400#, 800#, and 2000#) and cleaned thoroughly with anhydrous ethanol under ultrasonic treatment to remove surface contaminants and grease. The 5083 Al alloy samples were then dried with an air blower. The HF-SiO_2_@CS@EP/ethanol suspension was uniformly sprayed onto the surface of the 5083 Al alloy using a W-71 spray gun at a pressure of 0.2 MPa. During the spraying process, strict control was maintained over the spraying pressure and distance between the sample and the spray gun. After the spray-coating, the samples were cured on a heating stage at 80 °C to obtain the HF-SiO_2_@CS@EP superhydrophobic coating. The preparation process for the coating on other substrates was conducted similarly to the method described above. A schematic illustration of the fabrication process is shown in Figure 1.

### 2.4. Characterizations of Wettability, Morphology, and Chemical Composition

The contact angle (CA) and sliding angle (SA) of the samples were measured using a contact angle goniometer (Dataphysics OCA25, Stuttgart, Germany) with a droplet volume of 5 μL employed for the tests. A high-speed camera (Photron FASTCAM Mini UX100, Tokyo, Japan) was used to observe and record the dynamic impact behavior of water droplets on both uncoated and coated 5083 Al alloy surfaces. Field emission scanning electron microscopy (FE-SEM, FEI Nova NanoSEM 450, Waltham, MA, USA) was utilized to examine the micro- and nano-scale surface morphologies. Energy dispersive spectroscopy (EDS, Oxford X-Max N50), X-ray photoelectron spectroscopy (XPS, Thermo Scientific Escalab 250Xi, Waltham, USA), and Fourier-transform infrared spectroscopy (FTIR, Thermo Scientific NICOLET iS10, Waltham, MA, USA) were performed to characterize the elemental composition and functional groups on the sample surface.

### 2.5. Electrochemical Measurements

A 3.5 wt.% NaCl aqueous solution and an electrochemical workstation (Ametek Parstat P4000+, USA) were used to evaluate the electrochemical behavior of the various samples, including electrochemical impedance spectroscopy (EIS) and potentiodynamic polarization curves. The electrochemical tests were conducted using a three-electrode system, with the uncoated/coated Al alloy samples serving as the working electrode, a platinum sheet as the counter electrode, and a Ag/AgCl electrode as the reference electrode. During the tests, the exposure area of the working electrode in the electrolyte solution was fixed at 1 cm^2^. The frequency range for EIS measurements was from 10^5^ to 10⁻^2^ Hz. The three-electrode system must reach a stable open circuit potential (OCP) prior to EIS testing. The range for potentiodynamic polarization curves was ±250 mV vs. OCP. The obtained EIS data were analyzed and fitted using Zsimpwin software (3.30 version) for equivalent circuit modeling. The corrosion inhibition efficiency (*η*) of the coating is calculated using the following equation [37,38]:

(1) $$\eta =\frac{I_{corr\;bare}-I_{corr\;coated}}{I_{corr\;bare}}\times 100\%$$

where $I_{corr\;coated}$ and $I_{corr\;bare}$ represent the corrosion current density (*I*_corr_) of the HF-SiO_2_@CS@EP superhydrophobic coating and bare Al alloy substrate, respectively.

### 2.6. Anti-Icing Tests

For anti-icing tests, a custom-made low-temperature platform was utilized to observe and record the freezing process of water droplets on uncoated and coated samples in a −10 °C environment. To facilitate color observation during the low-temperature freezing experiments, methylene blue-stained water droplets were used for testing.

## 3. Results and Discussion

### 3.1. Surface Wettability

**Figure 2:**
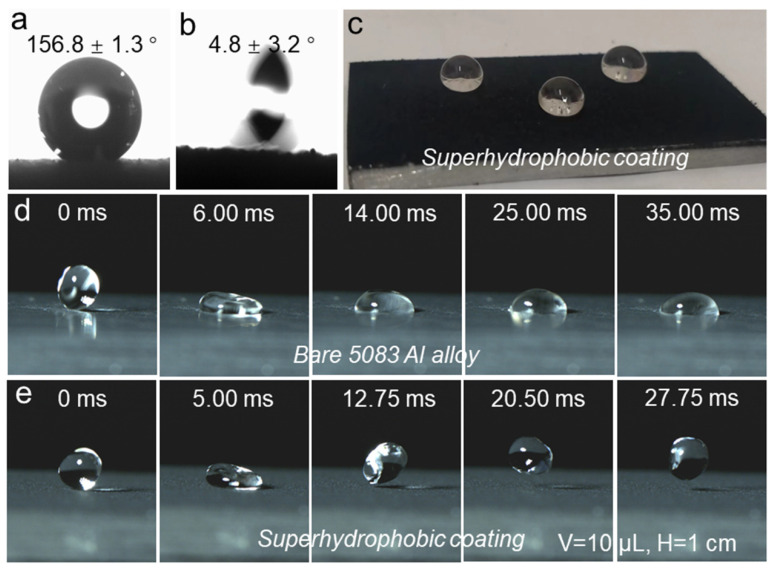
(**a**) CA image, (**b**) SA image, (**c**) optical image of water droplets placed on the coated sample, dynamic water droplet impact process on (**d**) bare 5083 Al alloy, and (**e**) superhydrophobic coating.

The images in Figure 2a,b illustrate the CA and SA of the coated 5083 Al alloy substrate. After applying the HF-SiO_2_@CS@EP coating, the CA and SA of the surface were measured as 156.8 ± 1.3° and 4.8 ± 3.2°, respectively, demonstrating superhydrophobic properties. The SA image indicates that the water droplet can easily roll off at very low tilt angles, which suggests a significantly reduced liquid–solid adhesion force on the surface. Figure 2c presents a macroscopic photo of water droplets positioned on the coated HF-SiO_2_@CS@EP superhydrophobic surface. Three 20 µL water droplets were gently placed on the coating, revealing that the droplets exhibit a typical Cassie–Baxter interfacial gas–liquid–solid contact state. The droplets maintained a spherical shape on the surface, indicating low surface energy and exceptional liquid-repellent properties.

To further compare the wettability changes in the 5083 Al alloy substrate before and after treatment, we utilized a high-speed camera to record the dynamic contact behavior of a 10 µL water droplet dropping from a height of 1 cm onto the surface, as shown in Figure 2d,e. Upon contact with the bare 5083 Al alloy substrate, the droplet quickly adhered and pinned to the surface, which can be attributed to the hydrophilic nature and higher surface energy of the uncoated Al alloy substrate. Conversely, for the HF-SiO_2_@CS@EP superhydrophobic coating, the water droplet exhibited a bouncing phenomenon after contacting the surface, where the first drop–bounce–release process took only 12.75 ms, demonstrating a remarkably short interface contact time along with strong non-wetting and liquid-repellent characteristics. These attributes collectively confirm the outstanding superhydrophobic characteristics and extremely low surface energy of our prepared coating.

### 3.2. Morphologies and Chemical Compositions

**Figure 3:**
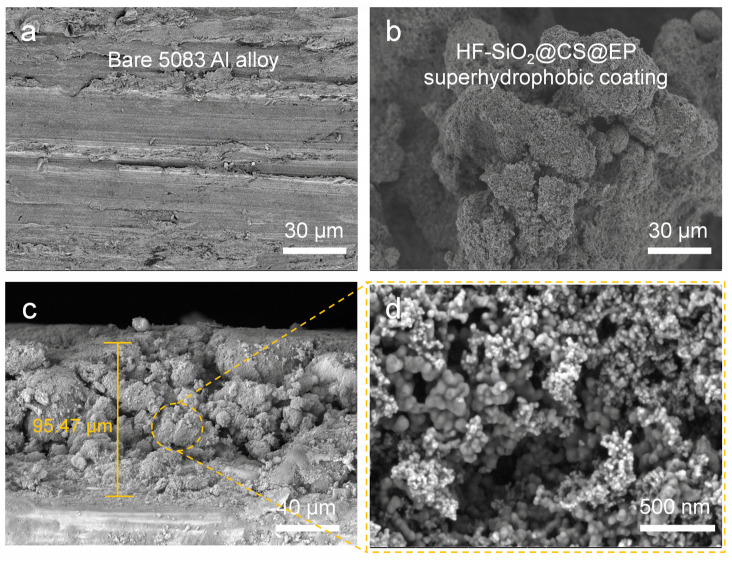
FE-SEM images of (**a**) bare 5083 Al alloy and (**b**) superhydrophobic coating; (**c**,**d**) cross-section SEM images of superhydrophobic coating.

Surface roughness and low surface energy are crucial for achieving superhydrophobic materials [39,40]. Figure 3 compares the surface morphology of the initial 5083 Al alloy substrate with that of the coated Al alloy substrate. Figure 3a displays the SEM image of the initial Al alloy, revealing a relatively smooth surface with only minor scratches from sanding. In contrast, Figure 3b shows the SEM image of the HF-SiO_2_@CS@EP superhydrophobic coating produced in this study, which exhibits irregular micron-sized protrusions resembling coral structures. The gaps between these protruding structures contain numerous voids, providing places for air entrapment. To further investigate the coating’s cross-section and thickness, we observed SEM cross-section images of the coated Al alloy by cutting the samples, as displayed in Figure 3c,d. The coating thickness was determined to be approximately 95.47 μm, revealing a fluctuating and uneven surface structure. At a higher magnification, a rich array of nano-scale structures was visible on the surface protrusions, which can be attributed primarily to the presence of HF-SiO_2_ and CS nanoparticles. The synergistic effect of the micro-/nano-binary structure significantly contributes to the achievement of excellent superhydrophobic performance. The composite morphology of micron structures along with abundant nano-scale features is highly beneficial for the stable retention of an air cushion, which is one of the critical conditions for forming the Cassie–Baxter contact state on the surface.

**Figure 4:**
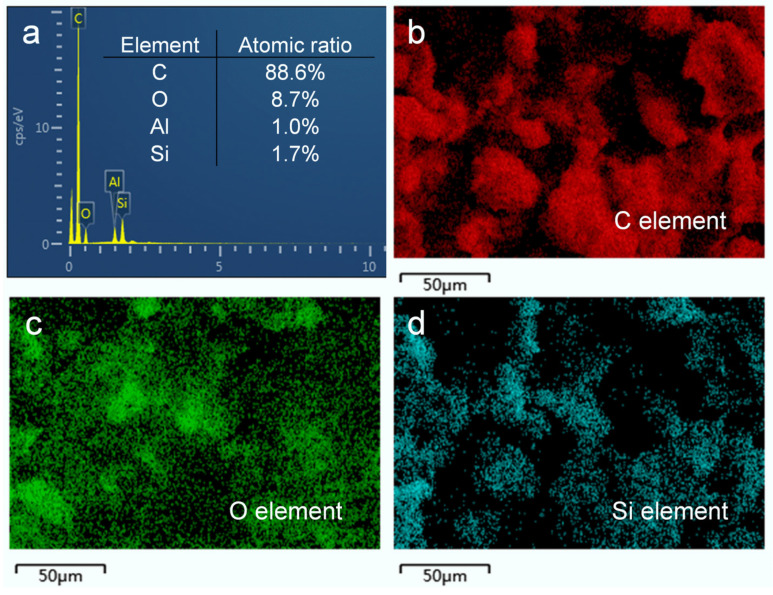
(**a**) EDS spectra and (**b**–**d**) EDS element mappings of C, O, and Si of the prepared superhydrophobic coating.

The analysis of the elemental composition and functional groups of the surface provides insights into the chemical composition of the prepared HF-SiO_2_@CS@EP superhydrophobic coating. In this study, we employed EDS, FTIR, and XPS to characterize the chemical compositions of the coating. Figure 4a shows the EDS spectrum of the HF-SiO_2_@CS@EP superhydrophobic coating, revealing that the surface is primarily composed of four elements: carbon (C), oxygen (O), aluminum (Al), and silicon (Si), with atomic percentages of 88.6%, 8.7%, 1.0%, and 1.7%, respectively. The high content of C observed can be attributed to the presence of CS, which is primarily made up of C nanoparticles exhibiting low surface energy characteristics. Figure 4b–d show elemental mappings of C, O, and Si, respectively. The maps indicate not only a pronounced uneven surface morphology but also a highly uniform distribution of elements across the surface. This uniform elemental distribution suggests that the prepared HF-SiO_2_@CS@EP superhydrophobic coating possesses excellent homogeneity, which is beneficial for achieving superhydrophobic properties.

**Figure 5:**
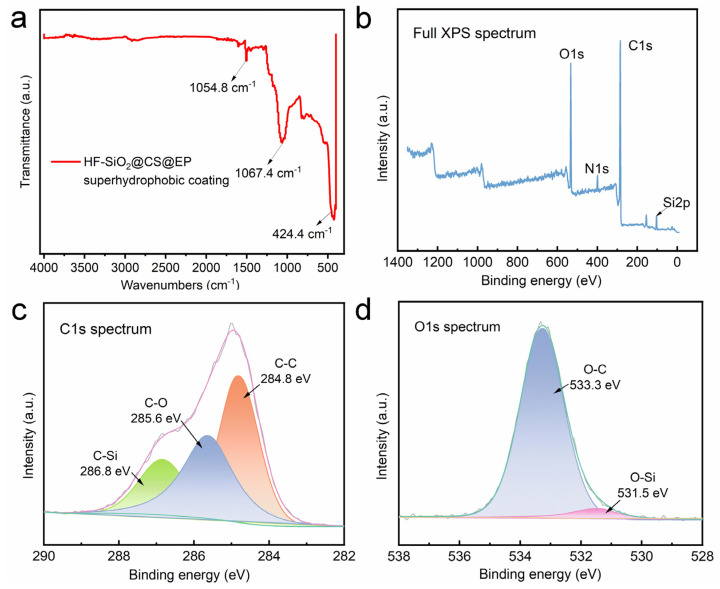
(**a**) FTIR spectra, (**b**) full XPS spectra, and high-resolution (**c**) C1s and (**d**) O1s spectra of the HF-SiO_2_@CS@EP superhydrophobic coating.

FTIR was utilized to investigate the chemical structure of the surface, as depicted in Figure 5a. A vibrational peak attributed to the stretching of Si-O bonds is observed at 1067.4 cm⁻^1^, stemming from the presence of HF-SiO_2_ molecules in the coating. The peak at 1054.8 cm⁻^1^ corresponds to the stretching vibrations of C=C bonds in aromatic rings, primarily due to the inclusion of epoxy resin within the coating. Additionally, the peak at 424.4 cm⁻^1^ can be assigned to C-C stretching vibrations because of CS. The analysis of these characteristic peaks confirms the successful homogeneous incorporation of HF-SiO_2_ and CS particles in the coating, along with the introduction of epoxy resin, resulting in the HF-SiO_2_@CS@EP superhydrophobic functional coating.

Figure 5b presents the full XPS spectrum of the HF-SiO_2_@CS@EP superhydrophobic coating, showing peaks corresponding to O1s, C1s, and Si2p. Figure 5c displays the high-resolution spectrum for C1s, revealing the binding energy positions for characteristic functional groups: C-Si, C-O, and C-C at 286.8 eV, 285.6 eV, and 284.8 eV, respectively. Figure 5d showcases the high-resolution spectrum for O1s, confirming the presence of O-C and O-Si groups, with binding energies located at 533.3 eV and 531.5 eV. The above characterization results regarding the surface chemical composition provide solid evidence that the produced HF-SiO_2_@CS@EP superhydrophobic coating comprises HF-SiO_2_ particles, CS particles, and epoxy resin. HF-SiO_2_ and CS particles contribute to the coating’s rich micro-/nano-structured morphology and its significantly enhanced roughness. Furthermore, the low surface energy properties of HF-SiO_2_ and CS particles greatly decrease the surface energy of the coating. The composite and uniform dispersion of these components is crucial for achieving the superhydrophobic performance highlighted in this study.

### 3.3. Anti-Corrosion Performance

**Figure 6:**
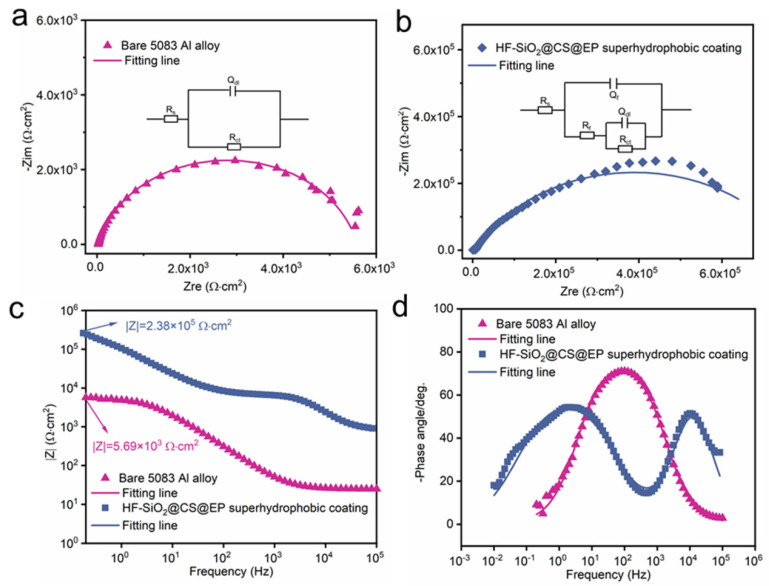
Nyquist plots of (**a**) bare 5083 Al alloy and (**b**) superhydrophobic coating-protected Al alloy. Bode plots of (**c**) frequency vs. modulus and (**d**) frequency vs. -phase angle.

EIS and potentiodynamic polarization curves serve as essential techniques for analyzing and comparing the corrosion resistance of materials [41,42,43]. The analysis of Nyquist and Bode plots allows for the observation of the electrochemical characteristics of different materials in corrosive media. Figure 6a,b depict the Nyquist plots of the uncoated and coated 5083 Al alloy in a 3.5 wt.% NaCl solution, respectively. It is evident that the capacitive arc radius of the HF-SiO_2_@CS@EP superhydrophobic coating-protected Al alloy substrate is significantly larger than that of the uncoated Al alloy. The inset images in Figure 6a,b represent the equivalent circuits of the bare 5083 Al alloy and the coated sample, respectively. Here, R_s_, R_f_, and R_ct_ denote the solution resistance, coating resistance, and charge transfer resistance, respectively. Q_f_ and Q_dl_ represent the film capacitance and double-layer capacitance, respectively. The fitted data are presented in Table 1, from which it can be seen that the R_ct_ value of the substrate protected by the HF-SiO_2_@CS@EP superhydrophobic coating has increased by two orders of magnitude. These findings highlight the significantly enhanced corrosion resistance properties conferred by the developed HF-SiO_2_@CS@EP superhydrophobic coating. Figure 6c,d present the frequency vs. modulus and frequency vs. -phase angle plots of the Bode plots. Typically, a higher modulus in the low-frequency region and a larger phase angle in the high-frequency region indicate better corrosion resistance of the material [44,45]. The low-frequency modulus for the bare Al alloy is 5.69 × 10^3^ Ω·cm^2^, while the low-frequency modulus for the coated Al alloy substrate is 2.38 × 10^5^ Ω·cm^2^, representing an increase of two orders of magnitude and demonstrating its excellent corrosion resistance.

**Figure 7:**
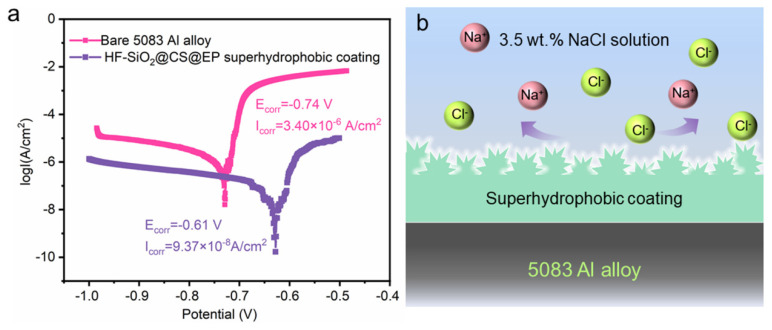
(**a**) Potentiodynamic polarization curves of the bare 5083 Al alloy and superhydrophobic coating-protected substrate. (**b**) Anti-corrosion mechanism of the superhydrophobic corrosion-resistant behaviors.

Furthermore, we conducted potentiodynamic polarization tests on both the bare and superhydrophobic samples, as shown in Figure 7a. The corrosion potential (*E*_corr_) and corrosion current (*I*_corr_) for the bare Al alloy are −0.74 V and 3.40 × 10^−6^ A/cm^2^, respectively. In contrast, the *E*_corr_ and *I*_corr_ for the HF-SiO_2_@CS@EP superhydrophobic coating are −0.61 V and 9.37 × 10^−8^ A/cm^2^, showing a positive shift of 130 mV in *E*_corr_ and a two-order-of-magnitude reduction in *I*_corr_. This indicates the coating’s outstanding corrosion protection effectiveness. Calculations based on Equation (1) reveal that the *η* of the HF-SiO_2_@CS@EP superhydrophobic coating-protected substrate is as high as 97.24%. Figure 7b illustrates the corrosion protection mechanism of the HF-SiO_2_@CS@EP superhydrophobic coating. When the Al alloy substrate covered with the HF-SiO_2_@CS@EP superhydrophobic coating is exposed to a corrosive medium, the surface non-wetting Cassie–Baxter state reduces the contact area with the corrosive liquid. Additionally, the air pockets trapped within the micro-/nano-hierarchical structures effectively enhance the surface charge transfer resistance, making it difficult for corrosive ions to diffuse and migrate on the surface. The excellent corrosion resistance and application potential of the superhydrophobic coating suggest a promising development landscape across various fields, including marine and industrial applications. However, a key research focus in the field that still remains is further enhancing the long-term corrosion resistance and interfacial stability of such materials.

### 3.4. Delayed Icing and Substrate Adaptability

**Figure 8:**
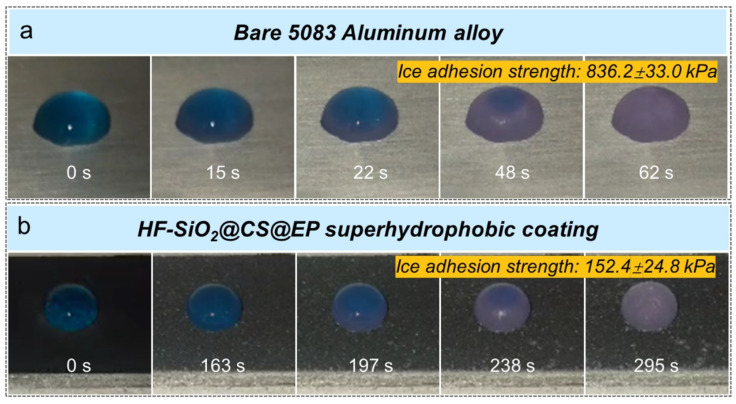
Water droplet icing process on (**a**) bare 5083 Al alloy and (**b**) superhydrophobic coating at −10 °C low-temperature environment.

Figure 8a,b show the results of water droplet freezing tests on the surfaces of a bare 5083 Al alloy and HF-SiO_2_@CS@EP superhydrophobic coating-protected substrate in a −10 °C low-temperature environment. Observations revealed that water droplets on the surface of the bare 5083 Al alloy exhibited hydrophilic behavior, completely freezing in 62 s. In contrast, water droplets on the Al alloy surface protected by the HF-SiO_2_@CS@EP superhydrophobic coating did not show significant freezing even after 163 s and were completely frozen after 295 s. The freezing time of dyed water droplets on the HF-SiO_2_@CS@EP superhydrophobic coated substrate was 4.75 times longer than that on the bare Al alloy substrate, extending the freezing time by 233 s. This indicates that the prepared HF-SiO_2_@CS@EP superhydrophobic coating exhibits outstanding delayed freezing performance. The delayed freezing characteristic primarily arises from the unique superhydrophobic properties of the surface significantly reducing the contact area between the liquid and the surface, thereby slowing the formation of ice nuclei. Additionally, the layer of air trapped in the surface structure further reduces the rate of thermal conduction.

Moreover, adhesion tests of the ice droplets to the surfaces after freezing indicate that the adhesion strength of ice on the bare Al alloy substrate is 836.2 ± 33.0 kPa, while that on the HF-SiO_2_@CS@EP superhydrophobic coating-protected surface is only 152.4 ± 24.8 kPa, which is merely one-fifth of that of the blank aluminum alloy substrate. The significant reduction in ice adhesion strength is attributed to the low surface energy of the HF-SiO_2_@CS@EP superhydrophobic coating, enhancing its anti-icing capability. Additionally, the markedly reduced ice adhesion allows for the possibility of ice removal from the surface under minimal mechanical force.

**Figure 9:**
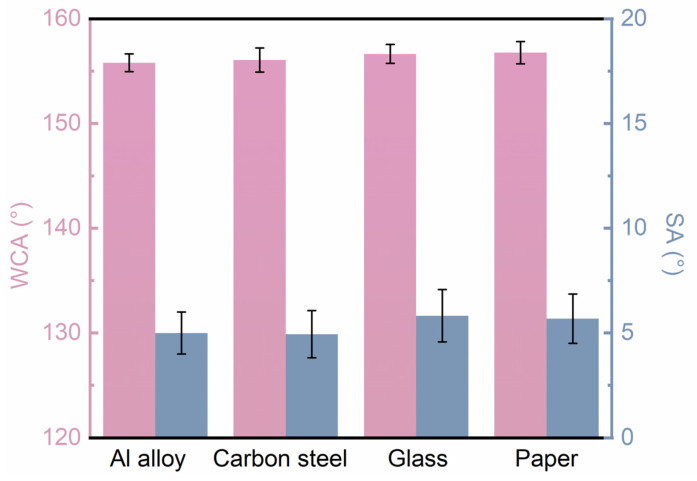
CA and SA data of the superhydrophobic coating prepared on different kinds of substrates.

Furthermore, to demonstrate the advantages of the coating’s applicability on different substrates, we prepared coatings not only on Al alloy but also on carbon steel, glass, paper, and various other substrates. The superhydrophobic properties of the coatings were evaluated through CA and SA measurements on the different substrates. Figure 9 presents the CA and SA data for the coatings on various substrates, showing that the CAs were all greater than 155° and the SAs were all less than 7°, indicating excellent superhydrophobic characteristics across different substrates. This confirms that the coating system developed in this study can be effectively applied using spraying techniques across a wide range of substrates. Coupled with its low cost and efficient production, the developed spraying method for superhydrophobic coatings holds promise as a reliable and functional protective material alternative for various materials across different industries.

## 4. Conclusions

In summary, this study employed a simple and easily applicable spraying method to construct a HF-SiO_2_@CS@EP superhydrophobic composite coating on the surface of 5083 Al alloys. The CA and SA of the prepared HF-SiO_2_@CS@EP superhydrophobic coating were measured at 156.8 ± 1.3° and 4.8 ± 3.2°, respectively. Investigation into the impact behavior of water droplets on the surface revealed that the prepared superhydrophobic coating facilitated rapid bouncing of the droplets, which further indicates that the surface possesses low surface energy and excellent liquid-repellent properties. An analysis of the surface morphology and chemical compositions revealed that the coating has a micro-/nano-binary structure, while the use of HF-SiO_2_ and CS particles contributes to the roughness and very low surface energy of the coating, which is a key factor in achieving superhydrophobicity. Additionally, the electrochemical testing results demonstrated that the charge transfer resistance and low-frequency modulus of the Al alloy substrate protected by the developed superhydrophobic coating significantly improved, with a positive shift in corrosion potential and a decrease of two orders of magnitude in corrosion current, showcasing a greatly enhanced corrosion protection capability. Moreover, the water droplet freezing experiments conducted in low-temperature environments further corroborated that the freezing time of the prepared superhydrophobic coating is significantly prolonged, with the ice adhesion strength being only one-fifth of that on the uncoated Al alloy substrate. These findings strongly indicate that superhydrophobic materials hold significant potential for development in various application areas.

## Figures and Tables

**Table 1:** The fitted EIS data of the bare 5083 Al alloy and HF-SiO_2_@CS@EP superhydrophobic coating.

Parameters	Bare 5083 Al Alloy	HF-SiO_2_@CS@EP Superhydrophobic Coating
R_S_ (Ω·cm^2^)	25.9	27.1
Q_f_ (Ω^−1^·cm^−2^·s^n1^)	/	1.11 × 10^−8^
n_1_	/	0.95
R_f_ (Ω·cm^2^)	/	5.70 × 10^3^
Q_dl_ (Ω^−1^·cm^−2^·s^n2^)	1.30 × 10^−5^	2.78 × 10^−6^
n_2_	0.86	0.70
R_ct_ (Ω·cm^2^)	6.05 × 10^3^	7.66 × 10^5^

## Data Availability

All data generated or analyzed during this study are included in this published article.
